# A possible mechanism of inhibition of U87MG and SH-SY5Y cancer cell proliferation by diallyl trisulfide and other aspects of its activity

**DOI:** 10.1007/s00726-017-2484-4

**Published:** 2017-08-29

**Authors:** Halina Jurkowska, Maria Wróbel, Marta Kaczor-Kamińska, Ewa Jasek-Gajda

**Affiliations:** 1 Chair of Medical Biochemistry, Jagiellonian University Medical College, 7 Kopernika St, 31-034 Kraków, Poland; 2 Department of Histology, Jagiellonian University Medical College, 7 Kopernika St, 31-034 Kraków, Poland

**Keywords:** Diallyl trisulfide, Sulfurtransferase, Sulfane sulfur, Glutathione, Cancer cells

## Abstract

The study was conducted to elucidate the mechanism of antiproliferative and antioxidative action of diallyl trisulfide (DATS), a garlic-derived organosulfur compound. Changes in the l-cysteine desulfuration, and the levels of cystathionine and non-protein thiols in DATS-treated human glioblastoma (U87MG) and neuroblastoma (SH-SY5Y) cells were investigated. The inhibition of proliferation of the investigated cells by DATS was correlated with an increase in the inactivated form of Bcl-2. In U87MG cells, an increased level of sulfane sulfur and an increased activity of 3-mercaptopyruvate sulfurtransferase (MPST) and rhodanese, the enzymes involved in sulfane sulfur generation and transfer, suggest that DATS can function as a donor of sulfane sulfur atom, transferred by sulfurtransferases, to sulfhydryl groups of cysteine residues of Bcl-2 and in this way lower the level of active form of Bcl-2 by *S*-sulfuration. Diallyl trisulfide antioxidative effects result from an increased level of cystathionine, a precursor of cysteine, and an increased glutathione level. MPST and rhodanese, the level of which is increased in the presence of DATS, can serve as antioxidant proteins.

## Introduction

Diallyl trisulfide (DATS), a sulfur compound derived from garlic, has various biological properties, such as anticancer (Liu et al. 2016; Pandrangi 2015; Ma et al. 2014; Hung et al. 2014; Shin et al. 2014; Zhou et al. 2009; Chandra-Kuntal et al. 2013; Wu et al. 2004; Xiao et al. 2004, 2006; Li et al. 2012; Shankar et al. 2008), antiangiogenic (Lai et al. 2015) and anti-inflammatory (Zhang et al. 2016; Kuo et al. 2013; Zeng et al. 2008) effects.

Diallyl trisulfide inhibits the growth of human cancer cells by inducing apoptosis in association with downregulation of Bcl-2 expression (Ma et al. 2014; Shin et al. 2014; Zhou et al. 2009; Li et al. 2012; Wan et al. 2013; Malki et al. 2009; Choi and Park 2012; Kim et al. 2007), induction of caspases and regulation of PI3 K/Akt and JNK pathways (Shin et al. 2014; Zhou et al. 2009; Choi and Park 2012; Seki et al. 2008; Borkowska et al. 2012). DATS-induced apoptosis of human pancreatic cancer cells is correlated with downregulation of Akt and cyclin D1 protein levels, and up-regulation of Bax, Fas, p53 and cyclin B protein levels (Ma et al. 2014). Recent studies have demonstrated the anticancer effects of DATS against breast cancer (Malki et al. 2009; Hahm and Singh 2014; Chandra-Kuntal et al. 2013; Nkrumah-Elie et al. 2012). DATS inhibits matrix metalloproteinases-2, and -9 (MMP2/9) activities and the metastasis of triple-negative breast cancer. The inhibitory effects are associated with downregulation of the transcriptional activities of NF-κB and ERK/MAPK signaling pathways (Liu et al. 2015). DATS suppresses the invasion of oral squamous cell carcinoma cell lines by reducing matrix MMP-9 via PI3K/AKT (Yang et al. 2012). Migration, invasion and angiogenesis of human colon cancer HT-29 cells and umbilical vein endothelial HUVEC cells are also inhibited by DATS (Lai et al. 2015). In HT29 cells, DATS inhibits migration and invasion through the inhibition of focal adhesion kinase (FAK), extracellular signal-regulated kinase, c-Jun N-terminal kinase and p38, which is associated with inhibition of MMP2/7/9 and VEGF. DATS affects inhibition of tumor growth, tumor weight and angiogenesis (decreasing the levels of hemoglobin) in HT29 cells (Lai et al. 2015). In human umbilical vein endothelial cells, DATS inhibits the migration and angiogenesis through FAK, Src and Ras; the secretion of VEGF is also inhibited in these cells.

Diallyl trisulfide demonstrates antioxidative effects, which are associated with the changes in the activity of antioxidant enzymes and the level of glutathione (Wu et al. 2004; Zhang et al. 2016; Zeng et al. 2008; Mostafa et al. 2016; Prabu and Sumedha 2014; Hu et al. 2007).

Diallyl trisulfide treatment resulted in increasing the levels of H_2_S (Chen et al. 2016; Tsai et al. 2015; Zhao et al. 2014). DATS reacts rapidly with reduced glutathione (GSH) to release H_2_S through thiol–disulfide exchange followed by allyl perthiol reduction by GSH (Liang et al. 2015). Benavides et al. (2007) first determined that DATS could be converted into H_2_S by human red blood cells or by rat aorta through a thiol, mainly glutathione, -dependent mechanism. H_2_S can be generated endogenously from l-cysteine by sulfurtransferases, including gamma-cystathionase (CTH), cystathionine beta-synthase (CBS), and 3-mercaptopyruvate sulfurtransferase (MPST) in combination with cysteine aminotransferase (Liu et al. 2016; Kolluru et al. 2013; Jurkowska et al. 2014). H_2_S can be oxidized to sulfane sulfur, a sulfur in the thiosulfoxide form (represented as S^0^), which plays important regulatory functions in biological systems (Toohey and Cooper 2014; Stein and Bailey 2013).

The study we have undertaken shows the effect of diallyl trisulfide on sulfane sulfur level, the activity of H_2_S-generating enzymes, the level of cystathionine and non-protein thiols, such as l-cysteine, L-cystine, and GSH and oxidized glutathione (GSSG), in human glioblastoma (U87MG) and neuroblastoma (SH-SY5Y) cells. We have shown that DATS results in an intensification of desulfuration pathways of L-cysteine by induction of sulfurtransferases activity, and causing an increase of the cystathionine and sulfane sulfur levels. Interestingly, we have found in this study that inhibition of U87MG and SH-SY5Y cells proliferation by DATS is also correlated with an increase in the inactivated form of Bcl-2 and the percentage of Bcl-2 non-expressing cells. Our results have confirmed (Jurkowska and Wróbel 2008; Jurkowska et al. 2011b) that inhibition of cell proliferation is correlated with elevation in intracellular sulfane sulfur level. The present results raise questions concerning the possible mechanism of inhibition of the Bcl-2 protein involved in the regulation of apoptosis. Our study has proven that DATS has an antioxidative effect on cancer cells by increasing the MPST and rhodanese activity and cystathionine and GSH levels.

## Materials and methods

### Sources of chemicals

Folin–Ciocialteau reagent, NADH, lactate dehydrogenase (LDH), pyridoxal phosphate (PLP), N-ethylmaleimide (NEM), bathophenanthrolinedisulfonic acid (BPDS), 2,4-dinitrofluorobenzene (DNFB), 1,4-dithio-bis-(2-nitrobenzoic acid) (DTT), acetonitrile, and crystal violet (N-hexamethylpararosaniline) were obtained from Sigma-Aldrich Corp. (St. Louis, MO, USA). Potassium cyanide (KCN) was obtained from Merck (Darmstadt, Germany), sodium 3-mercaptopyruvate, trifluoroacetic acid (TFA), and 2-mercaptoethanol from Flucka Chemie GmbH. Nε-methyl-l-lysine was purchased from Bachem (Bubendorf, Switzerland). Fetal bovine serum, trypsin, and penicillin/streptomycin were obtained from HyClone Laboratories (Utah, USA). The Cytotoxicity Detection Kit (LDH) was obtained from Roche Applied Science. All the other chemicals were of reagent grade and purchased from common commercial suppliers.

Diallyl trisulfide (DATS) was purchased from Cayman Chemical Company (Michigan, USA) and dissolved in dimethyl sulphoxide (DMSO; Sigma-Aldrich Corp., St. Louis, MO, USA), and then diluted with the medium Dulbecco’s Modified Eagle’s Medium (DMEM; HyClone Laboratories, Utah, USA) to the desired concentration prior to its use (the final concentration of DMSO in the medium was less than 0.1%).

### Cell culture

Human U87MG (glioblastoma) and SH-SY5Y (neuroblastoma) cells were obtained from the European Collection of Cell Cultures (ECACC) and maintained at 37 °C in humidified 95% air and 5% CO_2_ in DMEM supplemented with 10% fetal bovine serum (FBS), 2 mM l-glutamine, and 1% penicillin–streptomycin (100 Units/ml penicillin and 100 μg/ml streptomycin).

### Cell homogenization

U87MG and SH-SY5Y cells were suspended in 0.1 M phosphate buffer pH 7.5, in the ratio of 1 × 10^6^ cells/0.04 ml of the buffer, and sonicated 3 × 5 s at 4 °C (Bandelin Sonoplus GM 70). After centrifugation at 4500*g* at 4 °C for 10 min, the supernatant was used for the determination of protein concentration, sulfane sulfur levels and the activity of MPST, CTH, and rhodanese. For GSH, GSSG, l-cysteine, L-cystine and cystathionine, the cells were suspended in 0.1 ml 10% perchloric acid/1 mM BPDS. The sediment was separated by centrifugation at 1600*g* for 10 min, and the supernatant was saved at −80 °C until used for RP-HPLC analyses.

### Enzyme assay

MPST activity was assayed according to the method of Valentine and Frankenfeld (1974), with some modifications as described by Wróbel et al. (2004). The enzyme activity is expressed as nmoles of pyruvate produced during 1-min incubation at 37 °C per 1 mg of protein. Rhodanese activity was assayed by the Sorbo’s method (1955), following a procedure described in Wróbel et al. (2004). The enzyme activity is expressed as nmoles SCN^−^ formed during 1 min incubation at 20 °C per 1 mg of protein. CTH activity was determined according to Matsuo and Greenberg (1958) as modified by Czubak et al. (2002). The enzyme activity is expressed as nmoles of α-ketobutyrate formed during 1-min incubation at 37 °C per 1 mg of protein.

### Determination of sulfane sulfur level

Sulfane sulfur was determined by the method of Wood (1987), based on cold cyanolysis and colorimetric detection of ferric thiocyanate complex ion, and protein was determined by the method of Lowry et al. (1951) using crystalline bovine serum albumin as a standard.

### Determination of GSH, GSSG, l-cysteine, L-cystine, and cystathionine levels

RP-HPLC (Reversed-Phase High-Performance/Pressure Liquid Chromatography) method was used to determine the levels of such metabolites as l-cysteine, L-cystine, GSH and GSSG, and cystathionine in the investigated cells based on the method of Dominick et al. (2001), with some modification as described by Bronowicka-Adamska et al. (2011).

### Determination of cell viability

The effect of diallyl trisulfide on cell viability was assessed by measuring the leakage of lactate dehydrogenase (LDH) from dead or dying cells using a Cytotoxicity Detection Kit (Roche) as described previously (Jurkowska et al. 2011b). The 100 µM concentration of DATS that yielded LDH leakage of less than 5% was used for the experiments.

### Cell proliferation

The cells were seeded on 96-well plates at a concentration of 1.2 × 10^3^ cells/well (U87MG cells) or 1.5 × 10^3^ cells/well (SH-SY5Y cells) in DMEM supplemented as reported above. Following 24-h incubation, the culture medium was replaced with 100 µl of complete medium with DMSO (as the control) or 100 µl of medium containing 100 µM DATS and the plates were cultured for 24 and 48 h. The modified crystal violet staining method (Gillies et al. 1986) was used to determine the effect of DATS on the cell proliferation. The absorbance was measured at 540 nm using an Epoch Microplate Spectrophotometer (BioTek).

### Bcl-2 expression assay

Bcl-2 expression was analyzed using a Muse™ Bcl-2 Activation Dual Detection Kit (Millipore, Billerica, MA, USA) according to the manufacturer’s instruction. The assay utilizes two directly conjugated antibodies, a phospho-specific anti-phospho-Bcl-2 (Ser70)-Alexa Fluor 555 and an anti-Bcl-2-PECy5 conjugated antibody to measure total levels of Bcl-2 expression.

Briefly, 1 × 10^5^ cells were harvested, washed twice with 1X PBS and fixed with Fixation Buffer for 5 min on ice. Following the washing step with PBS, the cells were resuspended in Permeabilization Buffer and incubated for 5 min on ice. After washing with PBS, the cells were resuspended in 1X Assay Buffer containing the antibody working cocktail solution and incubated for 30 min in the dark at room temperature. The cells were analyzed by a Muse™ Cell Analyzer and a Muse™ analysis software (Millipore).

### Isolation of total RNA

Total RNA was extracted from the cells using TRIzol reagent (Invitrogen, CA, USA), according to the protocol provided by the manufacturer. The extracted RNA was suspended in ribonuclease-free water and was quantified by measuring the absorbance at 260 nm. The integrity of the purified RNA collected by this method was confirmed by observation of the 28S and 18S rRNA bands after agarose gel electrophoresis.

### Reverse transcription of RNA

Total RNA from the cell samples was reverse-transcribed using the GoScript™ Reverse Transcriptase according to the manufacturer’s instructions (Promega Corporation). For reverse transcription (RT), 3 µg of total RNA was mixed with 1 µl of Oligo d(*T*) primer (0.5 µg/μl) and water pretreated with diethylpyrocarbonate (H_2_O-DEPC) and incubated for 5 min at 70 °C. After preincubation, other components were added to this mixture: 4 µl GoScript™ 5× Reaction buffer (Promega Corporation), 3 µl MgCl_2_, 1 µl RNase inhibitor (20 U/µl), 1 µl deoxyribonucleotide triphosphates (dNTPs, 10 mM), and 1 µl GoScript™ Reverse Transcriptase (160 U/µl) in a total volume of 20 µl. The mixture was first incubated for 5 min at 25 °C, then for 60 min at 42 °C, and for the final 15 min at 70 °C, and stored at −20 °C.

### Polymerase chain reaction

Expressions of MPST, CTH, and β-actin were analyzed by PCR. Amplification of cDNA samples was run in a 25 µl reaction volume that contained the following: 2 µl of synthesized cDNA, 0.2 µM of each of gene-specific primer pair, 0.04 U/µl DNA polymerase in 10 mM buffer Tris– HCl pH 8.8 (supplemented with 1.5 mM MgCl_2_, 50 mM KCl, 0.1% Triton X-100), 0.2 mM of each dNTPs and H_2_O-DEPC.

For the MPST gene, PCR cycling conditions were 94 °C (5 min) for one cycle, 94 °C (30 s), 56 °C (30 s), and 72 °C (2 min) for 28 cycles, with a final extension 72 °C (8 min). Primer sequences were as follows: forward 5′ CCAGGTACCGTGAACATCCC 3′, and reverse 5′ATGTACCACTCCACCCAGGA 3′ (227 bp). The MPST mRNA sequence was obtained from NCBI. These PCR conditions for the MPST gene are published for the first time in this paper.

For the CTH gene, after an initial 5 min at 94 °C denaturation, amplification was performed under the following conditions: 94 °C for 30 s, 51 °C for 60 s, and 72 °C for 8 min for 28 cycles, with a final incubation at 72 °C for 10 min (Jurkowska et al. 2011a). The primer sequences were as follows: forward 5′-GCAAGTGGCATCTGAATTTG-3′, and reverse 5′-CCCATTACAACATCACTGTGG-3′ (301 bp) (Levonen et al. 2000).

For the β-Actin gene, after an initial 5 min at 94 °C denaturation, amplification was performed under the following conditions: 94 °C for 30 s, 54 °C for 30 s, and 72 °C for 2 min for 30 cycles, with a final incubation at 72 °C for 8 min (Jurkowska et al. 2011a). The primer sequences were as follows: forward 5′-CTGTCTGGCGGCACCACCAT-3′, and reverse 5′-GCAACTAAGTCATAGTCCGC-3′ (~300 bp) (Kusukawa et al. 2000).

β-Actin was used as an internal standard to normalize all the samples for potential variations in mRNA content. All PCR products were analyzed by electrophoresis on 2.0% agarose gel stained with ethidium bromide, and directly visualized under UV light and photographed.

### Statistical analysis

All the experiments were repeated at least three times. The data are expressed as mean ± standard deviation (SD). The statistical analysis was performed using the Student’s *t* test; values of *p < 0.05 were considered to indicate statistical significance.

## Results

### Effect of diallyl trisulfide on the proliferation of human glioblastoma (U87MG) and neuroblastoma (SH-SY5Y) cell lines

**Fig. 1:**
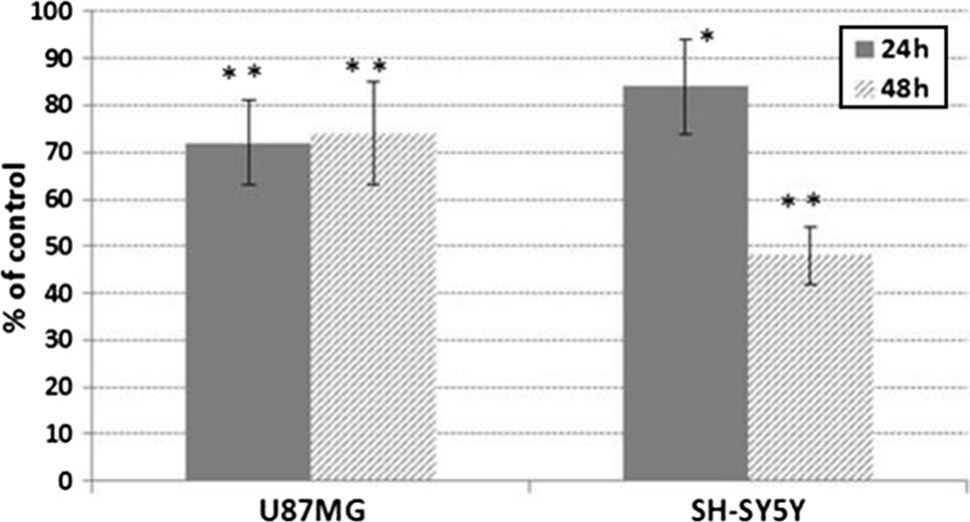
Effect of diallyl trisulfide on U87MG and SH-SY5Y cells proliferation. The cells were treated with 100 µM DATS for 24 and 48 h. The results are represented as a percentage of absorbance relative to the control cells (100%). Data represent mean ± SD; a statistical significance was shown as follows: *p < 0.01; **p < 0.001 (Student’s *t* test)

To examine the effects of DATS on the proliferation of human cancer cells, U87MG and SH-SY5Y cells were treated with 100 µM DATS and then subjected to crystal violet assay. As shown in Fig. 1, the growth of DATS-treated U87MG and SH-SY5Y cells was significantly decreased. After 24 h of culture, U87MG and SH-SY5Y cells proliferation decreased to about 72 and 84%, respectively, as compared to the control group. After 48 h of culture, DATS inhibited the growth of U87MG and SH-SY5Y cells to about 74 and 48%, respectively.

### Effect of diallyl trisulfide on the Bcl-2 expression in human glioblastoma (U87MG) and neuroblastoma (SH-SY5Y) cell lines

**Fig. 2:**
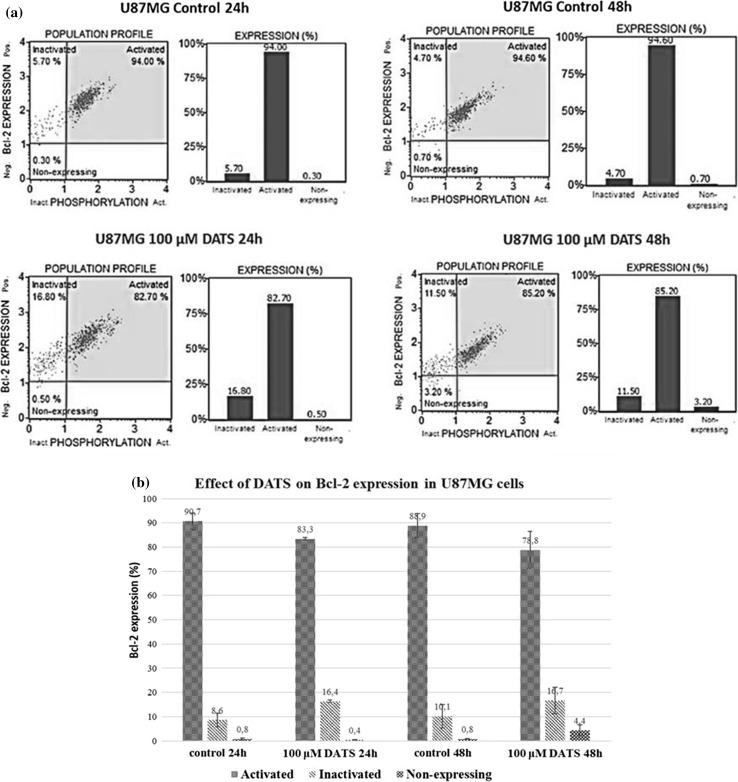
Effect of diallyl trisulfide on expression of Bcl-2 in U87MG cells. The cells were treated with 100 µM DATS for 24 and 48 h. Bcl-2 expression was analyzed using a Muse™ Bcl-2 Activation Dual Detection Kit. The samples were analyzed by flow cytometry. **a** One set of representative results is shown. **b** Each point represents the mean ± SD of three independent experiments

**Fig. 3:**
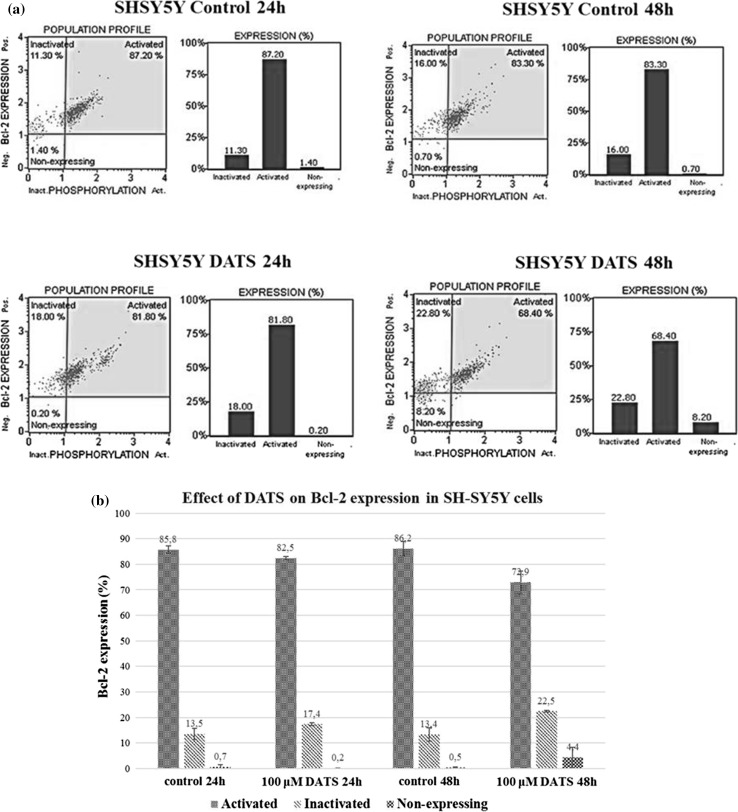
Effect of diallyl trisulfide on expression of Bcl-2 in SH-SY5Y cells. The cells were treated with 100 µM DATS for 24 and 48 h. Bcl-2 expression was analyzed using a Muse™ Bcl-2 Activation Dual Detection Kit. The samples were analyzed by flow cytometry. **a** One set of representative results is shown. **b** Each point represents the mean ± SD of three independent experiments

A Muse™ Bcl-2 Activation Dual Detection Kit was used to measure the percentage of Bcl-2 protein activation in SH-SY5Y and U87MG cells. Figures 2 and 3 show the percentage of cells with active and inactive (phosphorylated) form of Bcl-2, and non-expressing cells. As shown in Figs. 2 and 3, diallyl trisulfide causes an increase in Bcl-2 inactivated in U87MG and SH-SY5Y cells; the active form of Bcl-2 is decreased in both cancer cell lines. Our results indicate that DATS induces apoptosis via suppression of anti-apoptotic Bcl-2.

### Effect of diallyl trisulfide on the sulfurtransferases activity in human glioblastoma (U87MG) and neuroblastoma (SH-SY5Y) cell lines

**Fig. 4:**
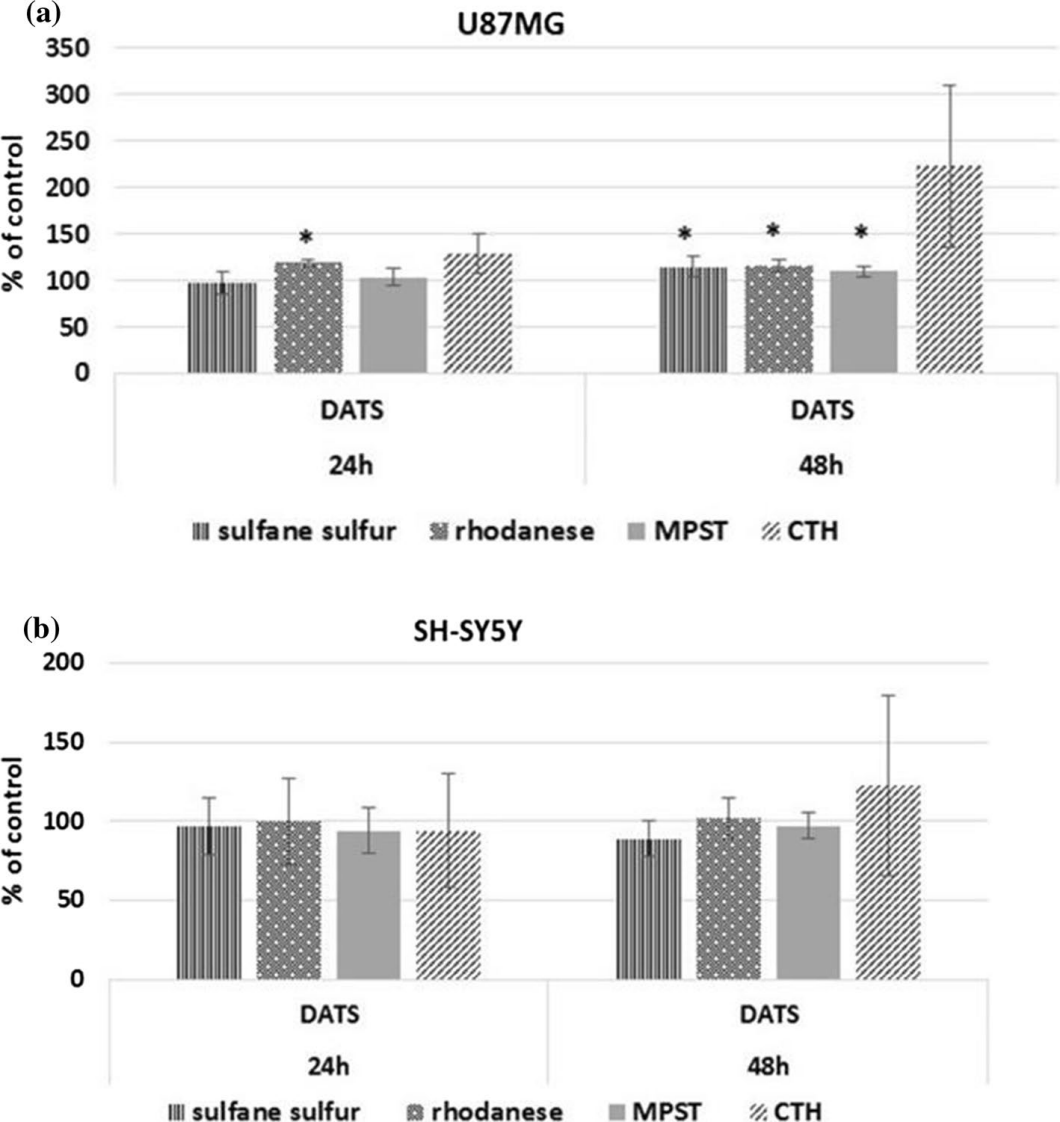
The effect of diallyl trisulfide on sulfurtransferases activity and sulfane sulfur levels in U87MG (**a**) and SH-SY5Y (**b**). The cells were treated with 100 µM DATS for 24 and 48 h. The values are mean ± SD from three independent experiments. In U87MG cells, rhodanese, MPST, and CTH activities determined after 24 h of culture equaled, respectively, 26 ± 1, 195 ± 13, and 1.2 ± 0.4 nmole/mg protein × min^−1^ (control values), and 31 ± 3, 208 ± 10, and 0.7 ± 0.3 nmole/mg protein × min^−1^ after 48 h (control values). Sulfane sulfur level determined after 24 and 48 h of culture equaled, respectively, 130 ± 20 and 134 ± 15 nmole/mg protein (control values). In SH-SY5Y cells, rhodanese, MPST, and CTH activities determined after 24 h of culture equaled, respectively, 67 ± 7, 889 ± 120, and 1.7 ± 0.7 nmole/mg protein × min^−1^ (control values), and 63 ± 4, 846 ± 44, and 1.3 ± 0.6 nmole/mg protein × min^−1^ after 48 h (control values). The sulfane sulfur level determined after 24 and 48 h of culture equaled, respectively, 134 ± 26, and 134 ± 23 nmole/mg protein (control values)

As shown in Fig. 4a, 100 µM DATS caused an elevation of the MPST activity, and the sulfane sulfur level in U87MG cells after 48 h of culture. The rhodanese activity was also increased in the presence of DATS after 24 and 48 h of culture (Fig. 4a). In SH-SY5Y cells, the activity of MPST and rhodanese, and the level of sulfane sulfur were not changed under these culture conditions (Fig. 4b). The activity of gamma-cystathionase was low in both cancer cell lines, and DATS did not cause a statistically significant difference in this enzyme activity (Fig. 4a, b).

### Effect of diallyl trisulfide on the expression of two sulfane sulfur and hydrogen sulfide generating sulfurtransferases in human glioblastoma (U87MG) and neuroblastoma (SH-SY5Y) cell lines

We did not observe statistically significant differences in the expression of mRNA of CTH and MPST genes in U87MG and SH-SY5Y cells after incubation with 100 µM DATS (not presented data).

### Effect of diallyl trisulfide on the level of non-protein thiols and cystathionine in human glioblastoma (U87MG) and neuroblastoma (SH-SY5Y) cell lines

The RP-HPLC method was used to investigate changes in the level of GSH, GSSG, l-cysteine, L-cystine, and cystathionine in U87MG and SH-SY5Y cells in the presence of diallyl trisulfide. In both cancer cell lines, an increased level of GSH and GSSG was detected after 24 and 48 h of culture in the presence of 100 µM DATS (Tables 1, 2). The intracellular level of l-cysteine in U87MG cells was higher (about 1 nmol/mg, control value) (Table 1) as compared to SH-SY5Y cells (not detected) (Table 2). In DATS-treated SH-SY5Y cells, the level of L-cystine was twofold increased as compared to the control cells (Table 2). In SH-SY5Y cells, the level of cystathionine was also increased in the presence of DATS (Table 2).

**Table 1:** DATS effect on the intracellular level of GSH, GSSG, l-cysteine, L-cystine, and cystathionine in U87MG cells

U87MG cells	GSH	GSSG	l-Cysteine	L-Cystine	Cystathionine
nmol/mg protein					
Control 24 h	21.9 ± 6.3	2.2 ± 0.7	1.0 ± 0.1	2.7 ± 0.5	0.05 ± 0.01
DATS 24 h	39.0 ± 2.9*	4.5 ± 0.1*	1.6 ± 0.4	2.5 ± 0.1	0.2 ± 0.1
Control 48 h	14.6 ± 2.8	1.5 ± 0.3	0.8 ± 0.1	1.4 ± 0.3	0.06 ± 0.01
DATS 48 h	26.7 ± 1.7*	2.8 ± 0.2*	1.2 ± 0.2	2.2 ± 0.3*	0.1 ± 0.03

The cells were incubated for 24 h and 48 h in the presence of 100 µM DATS. Every value represents the mean ± SD of three to five independent experiments

* p < 0.05 (Student’s *t* test)

**Table 2:** DATS effect on the intracellular level of GSH, GSSG, l-cysteine, L-cystine, and cystathionine in SH-SY5Y cells

SH-SY5Y cells	GSH	GSSG	L-Cysteine	L-Cystine	Cystathionine
nmol/mg protein					
Control 24 h	2.3 ± 0.3	1.0 ± 0.1	ND	0.9 ± 0.05	0.7 ± 0.1
DATS 24 h	7.5 ± 1.8*	1.8 ± 0.5*	ND	2.6 ± 0.1*	2.0 ± 0.1*
Control 48 h	2.3 ± 0.1	1.0 ± 0.1	ND	1.1 ± 0.02	0.7 ± 0.04
DATS 48 h	7.0 ± 0.9*	2.2 ± 0.2*	ND	2.0 ± 0.2*	1.9 ± 0.1*

The cells were incubated for 24 h and 48 h in the presence of 100 µM DATS. Every value represents the mean ± SD of three to five independent experiments. The level of L-cysteine was not detected (ND) in these cells

* *p* < 0.05 (Student’s *t* test)

## Discussion

### Anticancer effect of diallyl trisulfide on U87MG and SH-SY5Y cells

Garlic-derived organosulfur compounds provide significant protection against carcinogenesis (Capasso 2013; Wallace et al. 2013). In this paper, we have demonstrated that DATS inhibits proliferation of human glioblastoma (U87MG) and neuroblastoma (SH-SY5Y) cells. Our results show once again a relationship existing between the level of sulfane sulfur and cell proliferation. From the results we can conclude that inhibition of U87MG cells growth (Fig. 1) in the presence of DATS, as well as other cysteine precursors, such as N-acetyl-l-cysteine (Jurkowska and Wróbel 2008) and D-ribose-l-cysteine (Jurkowska et al. 2011b), is closely associated with an elevated intracellular sulfane sulfur level (Fig. 4a). Studies carried out by Predmore et al. (2012a, b) in mouse models of myocardial ischemia–reperfusion injury indicated that H_2_S and sulfane sulfur levels in the DATS-treated group were also significantly higher than those in the vehicle-treated group.

**Fig. 5:**
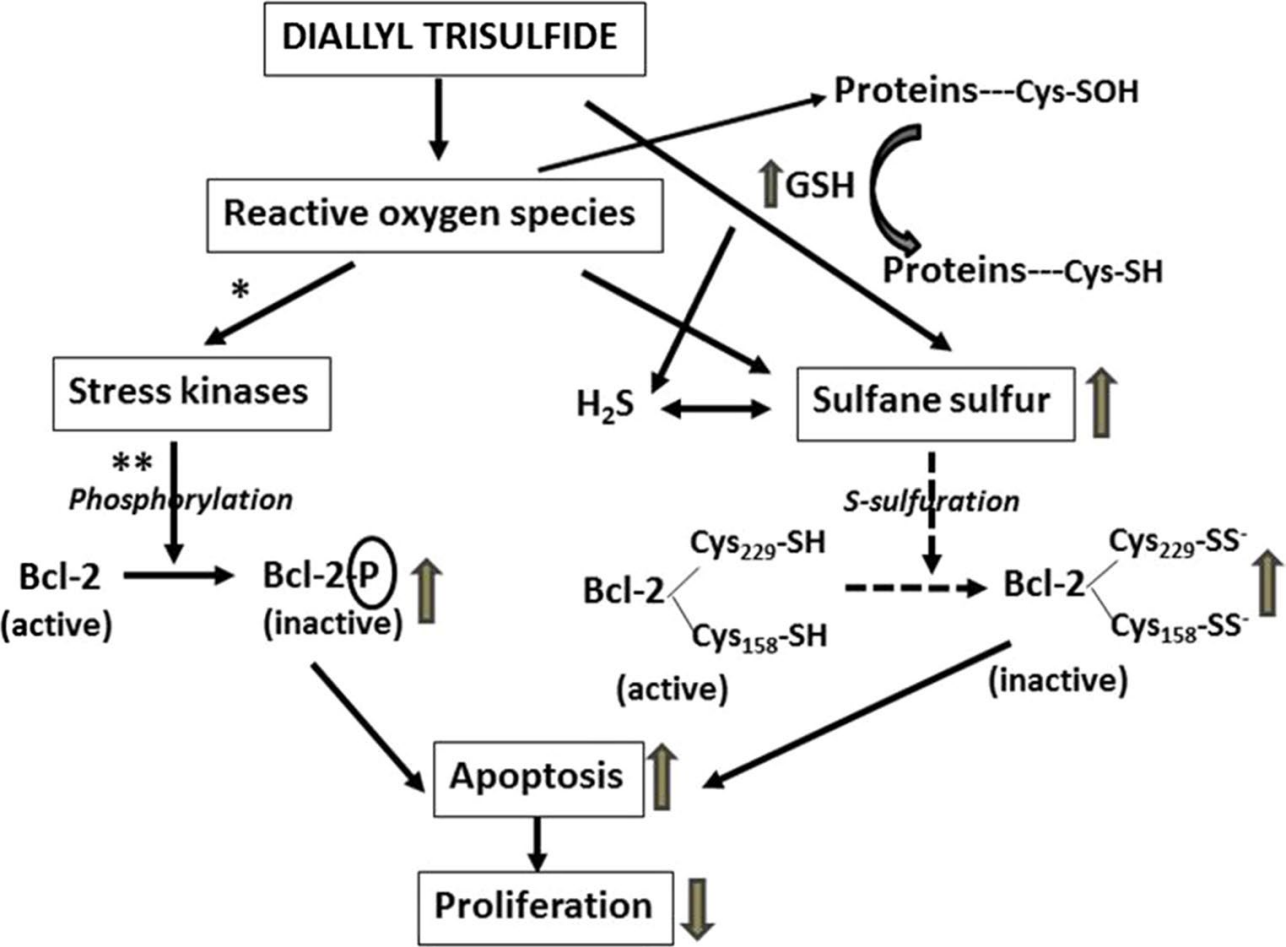
Suggested mechanisms of inhibition of U87MG and SH-SY5Y cells proliferation by diallyl trisulfide. *These reactions were confirmed by Das et al. (2007) and by **Xiao et al. (2004). The dashed lines show the suggested reactions

Furthermore, our results show that in U87MG as well as SH-SY5Y cells, DATS-induced inhibition of proliferation is associated with inactivation (phosphorylation) of Bcl-2 (Fig. 2). Previous studies indicate downregulation of Bcl-2 in the presence of DATS in human pancreatic (Ma et al. 2014), epithelial ovarian (Wan et al. 2013), leukemia (Choi and Park 2012), lung (Li et al. 2012), breast (Malki et al. 2009), and prostate (Kim et al. 2007) cancer cell lines. Bcl-2 protein contains four Bcl-2 homology domains with two cysteine residues at position 158 in the α5 domain and position 229 in the carboxyl-terminal membrane anchor domain (Cys-158 and Cys-229) (Luanpitpong et al. 2013). Azad et al. (2006) demonstrated that the two cysteine residues of Bcl-2 were important in the *S*-nitrosylation. Thus, it appears that modification of the Bcl-2 cysteine residues via S-sulfuration (–SH→–SSH) (Fig. 5) might be possible, especially in U87MG cells, in which DATS induces an increase in the MPST activity (enzyme responsible for the production and transfer of sulfane sulfur atoms) and sulfane sulfur level (Fig. 4a). Further studies will allow for explaining whether such a modification of Bcl-2 might be associated with the observed drop in Bcl-2 activity (Fig. 2) and inhibition of U87MG cells proliferation (Fig. 1).

The observed inhibition of SH-SY5Y cells proliferation by DATS (Fig. 1) can result from production of reactive oxygen species (ROS). In SH-SY5Y cells, in which the intracellular level of l-cysteine is very low (Table 2), inactivation (phosphorylation) of Bcl-2 caused by DATS could be probably associated with ROS production and activation of stress kinases (Fig. 5). Das et al. (2007) showed that garlic compounds (diallyl sulfide, diallyl disulfide) induced apoptosis in glioblastoma cells due to production of ROS, an increase in endoplasmic reticulum stress, a decrease in mitochondrial membrane potential, and activation of stress kinases and cysteine proteases. Treatment of glioblastoma cells with diallyl sulfide and diallyl disulfide induced an increase in phosphorylation of p38 MAPK and caused apoptosis (Das et al. 2007). Xiao et al. (2004) demonstrated that DATS-induced apoptosis in prostate cancer (PC-3) cells was associated with activation of extracellular signal-regulated kinase 1/2 (ERK1/2) and c-jun N-terminal kinase 1 (JNK1) and/or JNK2. Phosphorylation of Bcl-2 reduced its interaction with Bax to trigger mitochondrial caspase cascade.

### Antioxidative effect of diallyl trisulfide on U87MG and SH-SY5Y cells

Diallyl trisulfide has antioxidant effect on U87MG and SH-SY5Y cells by increasing the level of cystathionine and the level of GSH (Tables 1, 2), the most important intracellular thiolic antioxidant. GSH is a major determinant of the thiol/disulfide redox state, and a critical regulator of immune function, cell senescence, apoptosis, and vital redox-sensitive signaling pathways. It is highly likely that H_2_S formation from sulfane sulfur requires GSH as both hydrogen and electron donor (Predmore et al. 2012a, b). It was reported that DATS led to an increase in glutathione peroxidase and thioredoxin reductase activity in human lung cancer A549 cells; the glutathione reductase activity was decreased (Hu et al. 2007). In rat primary hepatocytes, DATS could enhance antioxidation and detoxification capabilities by increasing the intracellular GSH level and the activity of glutathione peroxidase, glutathione reductase, or glutathione S-transferase (Wu et al. 2004). DATS reduced levels of malondialdehyde, asymmetric dimethylarginine, and acetylcholinestrase activity, while increasing GSH levels (Mostafa et al. 2016). DATS inhibited oxidative stress and apoptosis in an ethanol-induced model (Chen et al. 2016), and had a protective effect against arsenic-induced oxidative stress in rat erythrocytes and lymphocytes (Prabu and Sumedha 2014). The levels of lipid peroxidation markers, such as thiobarbituric acid reactive substances, malondialdehyde, lipid hydroperoxides, conjugated dienes and protein carbonyl, were significantly decreased and there was a significant increase in ATPase activities and non-enzymatic and enzymatic antioxidants on treatment with DATS in a dose-related manner (Prabu and Sumedha 2014).

Our results confirm the antioxidant properties of diallyl trisulfide. It induces rhodanese and MPST activity in U87MG cells (Fig. 4a). The rhodanese participates in sulfane sulfur metabolism (Ubuka et al. 2008) and is one of the enzymes able to catalyze H_2_S formation, in the presence of thiosulfate and dithiothreitol (Mikami et al. 2011). MPST is a protein closely related to rhodanese (Nakajima 2015). Rhodanese, similarly to MPST, can generate H_2_S reacting with thioredoxin (Mikami et al. 2011; Yadav et al. 2013). Thus, rhodanese could be involved in both the metabolism of organosulfur compounds and in the production of H_2_S in mitochondria (Bhuiyan et al. 2015). Since it has also been demonstrated that MPST has a role in anti-oxidative defense systems (Nagahara et al. 2007, 2013), both MPST and rhodanese could also serve as antioxidant proteins (Nakajima 2015). When MPST and rhodanese are oxidized, catalytic site cysteines are reversibly converted to sulfenyl (Nagahara 2011). Stable and low redox sulfenate is formed and then is reduced by thioredoxin (Nagahara et al. 2007, 2013; Nagahara 2011). Under oxidizing conditions, the cysteine pool is increased because of post-translational regulation of methionine synthase (Mosharov et al. 2000), cystathione β-synthase (Taoka et al. 1998) and MPST (Nagahara et al. 2015; Nagahara and Katayama 2005). An increase in the cysteine content in the cell results in an increase in the content of cellular reductants, such as thioredoxin and glutathione. Thus, MPST and rhodanese serve as antioxidant proteins and partly maintain cellular redox homeostasis (Nagahara 2011). Krueger et al. (2010) demonstrated that the reduction of rhodanese expression indicated an increase of oxidative stress and predicted mortality in hemodialysis patients. Additionally, administration of sodium thiosulfate, a substrate of rhodanese, prevented acute inflammatory liver failure by augmenting thiosulfate levels and upregulating antioxidant and anti-apoptotic defense in the liver (Shirozu et al. 2014).

## Conclusions

Diallyl trisulfide inhibits proliferation of U87MG and SH-SY5Y cancer cells. In U87MG cells, in the presence of an increased activity of MPST and rhodanese and an increased sulfane sulfur level, the sulfhydryl groups of Bcl-2 can be modified via S-sulfuration (Fig. 5). Through its effect on inactivation of Bcl-2, the modification can inhibit proliferation of these cells. In SH-SY5Y cells, where cysteine level is negligible, inactivation of Bcl-2 protein following DATS administration can result from an increased level of reactive oxygen species.

The antioxidative potential of DATS is supported by an elevated level of glutathione and cystathionine, as well as an increased activity of MPST and rhodanese in the cells.

## Abbreviations

DATS: Diallyl trisulfide
U87MG: The human glioblastoma cell line
SH-SY5Y: The human neuroblastoma cell line
CBS: Cystathionine-β-synthase
CTH: Cystathionine-γ-lyase
GSH: Reduced glutathione
GSSG: Oxidazed glutathione
Cys: l-cysteine
RP-HPLC: Reversed-phase high-performance liquid chromatography
MPST: 3-Mercaptopyruvate sulfurtransferase
Bcl-2: B-cell lymphoma-2
PI3 K: Phosphoinositide 3-kinase
AKT: Serine/threonine-specific protein kinase
JNK: C-jun N-terminal kinase
MMP: Metalloproteinase
ERK: Extracellular signal-regulated kinases
MAPK: Mitogen-activated protein kinases
VEGF: Vascular endothelial growth factor
FAK: Focal adhesion kinase
Src: Protein tyrosine kinase
HT29: The human colon cancer cell line

## References

[CR1] Azad N, Vallyathan V, Wang L, Tantishaiyakul V, Stehlik C, Leonard SS, Rojanasakul Y (2006). S-nitrosylation of Bcl-2 inhibits its ubiquitin-proteasomal degradation. A novel antiapoptotic mechanism that suppresses apoptosis. J Biol Chem.

[CR2] Benavides GA, Squadrito GL, Mills RW, Patel HD, Isbell TS, Patel RP, Darley-Usmar VM, Doeller JE, Kraus DW (2007). Hydrogen sulfide mediates the vasoactivity of garlic. Proc Natl Acad Sci USA.

[CR3] Bhuiyan AI, Papajani VT, Paci M, Melino S (2015). Glutathione-garlic sulfur conjugates: slow hydrogen sulfide releasing agents for therapeutic applications. Molecules.

[CR4] Borkowska A, Sielicka-Dudzin A, Herman-Antosiewicz A, Wozniak M, Fedeli D, Falcioni G, Antosiewicz J (2012). Diallyl trisulfide-induced prostate cancer cell death is associated with Akt/PKB dephosphorylation mediated by Pp66shc. Eur J Nutr.

[CR5] Bronowicka-Adamska P, Zagajewski J, Czubak J, Wróbel M (2011). RP-HPLC method for quantitative determination of cystathionine, cysteine and glutathione: an application for the study of the metabolism of cysteine in human brain. J Chromatogr B Analyt Technol Biomed Life Sci.

[CR6] Capasso A (2013). Antioxidant action and therapeutic efficacy of Allium sativum L. Molecules.

[CR7] Chandra-Kuntal K, Lee J, Singh SV (2013). Critical role for reactive oxygen species in apoptosis induction and cell migration inhibition by diallyl trisulfide, a cancer chemopreventive component of garlic. Breast Cancer Res Treat.

[CR8] Chen LY, Chen Q, Zhu XJ, Kong DS, Wu L, Shao JJ, Zheng SZ (2016). Diallyl trisulfide protects against ethanol-induced oxidative stress and apoptosis via a hydrogen sulfide-mediated mechanism. Int Immunopharmacol.

[CR9] Choi YH, Park HS (2012). Apoptosis induction of U937 human leukemia cells by diallyl trisulfide induces through generation of reactive oxygen species. J Biomed Sci.

[CR10] Czubak J, Wróbel M, Jurkowska H (2002). Cystathionine γ-lyase (EC 4.4.1.1): an enzymatic assay of α-ketobutyrate using lactate dehydrogenase. Acta Biol Crac Ser Zool.

[CR11] Das A, Banik NL, Ray SK (2007). Garlic compounds generate reactive oxygen species leading to activation of stress kinases and cysteine proteases for apoptosis in human glioblastoma T98G and U87MG cells. Cancer.

[CR12] Dominick PK, Cassidy PB, Roberts JC (2001). A new and versatile method for determination of thiolamines of biological importance. J Chromatogr B Biomed Sci Appl.

[CR13] Gillies RJ, Didier N, Denton M (1986). Determination of cell number in monolayer cultures. Anal Biochem.

[CR14] Hahm ER, Singh SV (2014). Diallyl trisulfide inhibits estrogen receptor-α activity in human breast cancer cells. Breast Cancer Res Treat.

[CR15] Hu Y, Urig S, Koncarevic S, Wu X, Fischer M, Rahlfs S, Mersch-Sundermann V, Becker K (2007). Glutathione- and thioredoxin related enzymes are modulated by sulfur-containing chemopreventive agents. Biol Chem.

[CR16] Hung FM, Shang HS, Tang NY, Lin JJ, Lu KW, Lin JP, Ko YC, Yu CC, Wang HL, Liao JC, Lu HF, Chung JG (2014). Effects of diallyl trisulfide on induction of apoptotic death in murine leukemia WEHI-3 cells in vitro and alterations of the immune responses in normal and leukemic mice in vivo. Environ Toxicol.

[CR17] Jurkowska H, Wróbel M (2008). N-acetyl-l-cysteine as a source of sulfane sulfur in astrocytoma and astrocyte cultures: correlations with cell proliferation. Amino Acids.

[CR18] Jurkowska H, Placha W, Nagahara N, Wróbel M (2011). The expression and activity of cystathionine-c-lyase and 3-mercaptopyruvate sulfurtransferase in human neoplastic cell lines. Amino Acids.

[CR19] Jurkowska H, Uchacz T, Roberts J, Wróbel M (2011). Potential therapeutic advantage of ribose-cysteine in the inhibition of astrocytoma cell proliferation. Amino Acids.

[CR20] Jurkowska H, Roman HB, Hirschberger LL, Sasakura K, Nagano T, Hanaoka K, Krijt J, Stipanuk MH (2014). Primary hepatocytes from mice lacking cysteine dioxygenase show increased cysteine concentrations and higher rates of metabolism of cysteine to hydrogen sulfide and thiosulfate. Amino Acids.

[CR21] Kim YA, Xiao D, Xiao H, Powolny AA, Lew KL, Reilly ML, Zeng Y, Wang Z, Singh SV (2007). Mitochondria-mediated apoptosis by diallyl trisulfide in human prostate cancer cells is associated with generation of reactive oxygen species and regulated by Bax/Bak. Mol Cancer Ther.

[CR22] Kolluru GK, Shen X, Bir SC, Kevil CG (2013). Hydrogen sulfide chemical biology: pathophysiological roles and detection. Nitric Oxide.

[CR23] Krueger K, Koch K, Juehling A, Tepel M, Scholze A (2010). Low expression of thiosulfate sulfurtransferase (rhodanese) predicts mortality in hemodialysis patients. Clin Biochem.

[CR24] Kuo WW, Wang WJ, Tsai CY, Way CL, Hsu HH, Chen LM (2013). Diallyl trisufide (DATS) suppresses high glucose-induced cardiomyocyte apoptosis by inhibiting JNK/NFkappaB signaling via attenuating ROS generation. Int J Cardiol.

[CR25] Kusukawa J, Suefuji Y, Ryu F, Noguchi R, Iwamoto O (2000). Kameyama, T. Dissemination of cancer cells into circulation occurs by incisional biopsy of oral squamous cell carcinoma. J Oral Pathol Med.

[CR26] Lai KC, Hsu SC, Yang JS, Yu CC, Lein JC, Chung JG (2015). Diallyl trisulfide inhibits migration, invasion and angiogenesis of human colon cancer HT-29 cells and umbilical vein endothelial cells, and suppresses murine xenograft tumour growth. J Cell Mol Med.

[CR27] Levonen AL, Lapatto R, Saksela M, Raivi KO (2000). Human cystathionine γ-lyase: developmental and in vitro expression of two isoforms. Biochem J.

[CR28] Li W, Tian H, Li L, Li S, Yue W, Chen Z, Qi L, Hu W, Zhu Y, Hao B, Gao C, Si L, Gao F (2012). Diallyl trisulfide induces apoptosis and inhibits proliferation of A549 cells in vitro and in vivo. Acta Biochim Biophys Sin.

[CR29] Liang D, Wu H, Wong MW, Huang D (2015). Diallyl trisulfide is a fast H_2_S donor, but diallyl disulfide is a slow one: the reaction pathways and intermediates of glutathione with polysulfides. Org Lett.

[CR30] Liu Y, Zhu P, Wang Y, Wei Z, Tao L, Zhu Z, Sheng X, Wang S, Ruan J, Liu Z, Cao Y, Shan Y, Sun L, Wang A, Chen W, Lu Y (2015). Antimetastatic therapies of the polysulfide diallyl trisulfide against triple-negative breast cancer (TNBC) via suppressing MMP2/9 by blocking NF-κB and ERK/MAPK signaling pathways. PLoS ONE.

[CR31] Liu M, Wu L, Montaut S, Yang G (2016). Hydrogen sulfide signaling axis as a target for prostate cancer therapeutics. Prostate cancer. dx..

[CR32] Lowry OH, Rosebrough NJ, Farr L, Randall RJ (1951). Protein measurement with the folin phenol reagent. J Biol Chem.

[CR33] Luanpitpong S, Chanvorachote P, Stehlik C, Tse W, Callery PS, Wang L, Rojanasakul Y (2013). Regulation of apoptosis by Bcl-2 cysteine oxidation in human lung epithelial cells. Mol Biol Cell.

[CR34] Ma HB, Huang S, Yin XR, Zhang Y, Di ZL (2014). Apoptotic pathway induced by diallyl trisulfide in pancreatic cancer cells. World J Gastroenterol.

[CR35] Malki A, El-Saadani M, Sultan AS (2009). Garlic constituent diallyl trisulfide induced apoptosis in MCF7 human breast cancer cells. Cancer Biol Ther.

[CR36] Matsuo Y, Greenberg DM (1958). A crystalline enzyme that cleaves homoserine and cystathionine: i. Isolation procedure and some physiochemical properties. J Biol Chem.

[CR37] Mikami Y, Shibuya N, Kimura Y, Nagahara N, Ogasawara Y, Kimura H (2011). Thioredoxin and dihydrolipoic acid are required for 3 mercaptopyruvate sulfurtransferase to produce hydrogen sulfide. Biochem J.

[CR38] Mosharov E, Cranford MR, Banerjee R (2000). The quantitatively important relationship between homocysteine metabolism and glutathione synthesis by the transsulfuration pathway and its regulation by redox changes. Biochemistry.

[CR39] Mostafa DK, El Azhary NM, Nasra RA (2016). The hydrogen sulfide releasing compounds ATB-346 and diallyl trisulfide attenuate streptozotocin-induced cognitive impairment, neuroinflammation, and oxidative stress in rats: involvement of asymmetric dimethylarginine. Can J Physiol Pharmacol.

[CR40] Nagahara N (2011) Catalytic site cysteines of thiol enzyme: sulfurtransferases. J Amino Acids, 709404 10.4061/2011/709404 PMC3276061 22332003

[CR41] Nagahara N, Katayama A (2005). Post-translational regulation of mercaptopyruvate sulfurtransferase via a low redox potential cysteine-sulfenate in the maintenance of redox homeostasis. J Biol Chem.

[CR42] Nagahara N, Yoshii T, Abe Y, Matsumura T (2007). Thioredoxin-dependent enzymatic activation of mercaptopyruvate sulfurtransferase – An intersubunit disulfide bond serves as a redox switch for activation. J Biol Chem.

[CR43] Nagahara N, Nagano M, Ito T, Shimamura K, Akimoto T, Suzuki H (2013). Antioxidant enzyme, 3-mercaptopyruvate sulfurtransferase-knockout mice exhibit increased anxiety-like behaviors: a model for human mercaptolactate-cysteine disulfiduria. Sci Rep.

[CR44] Nagahara N, Nagano M, Ito T, Suzuki H (2015). Redox regulation of mammalian 3-mercaptopyruvate sulfurtransferase. Methods Enzymol.

[CR45] Nakajima T (2015). Roles of sulfur metabolism and rhodanese in detoxification and anti-oxidative stress functions in the liver: responses to radiation exposure. Med Sci Monit.

[CR46] Nkrumah-Elie YM, Reuben JS, Hudson A, Taka E, Badisa R, Ardley T, Israel B, Sadrud-Din SY, Oriaku E, Darling-Reed SF (2012). Diallyl trisulfide as an inhibitor of benzo(a)pyreneinduced precancerous carcinogenesis in MCF-10A cells. Food Chem Toxicol.

[CR47] Pandrangi A (2015). Cancer Chemoprevention by Garlic - A Review Hereditary Genet.

[CR48] Prabu SM, Sumedha NC (2014). Ameliorative effect of diallyl trisulphide on arsenic-induced oxidative stress in rat erythrocytes and DNA damage in lymphocytes. J Basic Clin Physiol Pharmacol.

[CR49] Predmore BL, Kondo K, Bhushan S, Zlatopolsky MA, King AL, Aragon JP, Grinsfelder DB, Condit ME, Lefer DJ (2012). The polysulfide diallyl trisulfide protects the ischemic myocardium by preservation of endogenous hydrogen sulfide and increasing nitric oxide bioavailability. Am J Physiol Heart Circ Physiol.

[CR50] Predmore BL, Lefer DJ, Gojon G (2012). Hydrogen sulfide in biochemistry and medicine. Antioxid Redox Signal.

[CR51] Seki T, Hosono T, Hosono-Fukao T, Inada K, Tanaka R, Ogihara J, Ariga T (2008). Anticancer effects of diallyl trisulfide derived from garlic. Asian Pac J Clin Nutr.

[CR52] Shankar S, Chen Q, Ganapathy S, Singh KP, Srivastava RK (2008). Diallyl trisulfide increases the effectiveness of TRAIL and inhibits prostate cancer growth in an orthotopic model: molecular mechanisms. Mol Cancer Ther.

[CR53] Shin DY, Kim G-Y, Hwang HJ, Kim WJ, Choi YH (2014). Diallyl trisulfide-induced apoptosis of bladder cancer cells is caspase-dependent and regulated byPI3 K/Akt and JNK pathways. Environ Toxicol Pharmacol.

[CR54] Shirozu K, Tokuda K, Marutani E (2014). Cystathionine γ-lyase deficiency protects mice from galactosamine/lipopolysaccharide induced acute liver failure. Antioxid Redox Signal.

[CR55] Stein A, Bailey SM (2013). Redox biology of hydrogen sulfide: implications for physiology, pathophysiology, and pharmacology. Redox Biol.

[CR56] Sӧrbo B (1955). Rhodanese. Methods Enzymol.

[CR57] Taoka S, Ohja S, Shan X, Kruger WD, Banerjee R (1998). Evidence for heme-mediated redox regulation of human cystathionine beta-synthase activity. J Biol Chem.

[CR58] Toohey JI, Cooper AJL (2014). Thiosulfoxide (sulfane) sulfur: new chemistry and new regulatory roles in biology. Molecules.

[CR59] Tsai CY, Wen SY, Shibu MA, Yang YC, Peng H, Wang B, Wei YM, Chang HY, Lee CY, Huang CY, Kuo WW (2015). Diallyl trisulfide protects against high glucose-induced cardiac apoptosis by stimulating the production of cystathionine gamma-lyase-derived hydrogen sulfide. Int J Cardiol.

[CR60] Ubuka T, Okada A, Nakamura H (2008). Production of hypotaurine from l-cysteinesulfinate by rat liver mitochondria. Amino Acids.

[CR61] Valentine WN, Frankenfeld JK (1974). 3-Mercaptopyuruvate sulfurtransferase (EC 2.8.1.2): a simple assay adapted to human blood cells. Clin Chim Acta.

[CR62] Wallace GC, Haar CP, Vandergrift WA, Giglio P, Dixon-Mah YN, Varma AK, Ray SK, Patel SJ, Banik NL, Das A (2013). Multi-targeted DATS prevents tumor progression and promotes apoptosis in ectopic glioblastoma xenografts in SCID mice via HDAC inhibition. J Neurooncol.

[CR63] Wan HF, Yu LH, Wu JL, Tu S, Zhu WF, Zhang XL, Wan FS (2013). Effect of diallyl trisulfide on human ovarian cancer SKOV- 3/DDP Cell Apoptosis. Asian Pac J Cancer Prev.

[CR64] Wood L (1987). Sulfane sulfur. Methods Enzymol.

[CR65] Wróbel M, Jurkowska H, Śliwa L, Srebro Z (2004). Sulfurtransferases and cyanide detoxification in mouse liver, kidney, and brain. Toxicol Mech Methods.

[CR66] Wu CC, Lii CK, Tsai SJ, Sheen LY (2004). Diallyl trisulfide modulates cell viability and the antioxidation and detoxification systems of rat primary hepatocytes. J Nutr.

[CR67] Xiao D, Choi S, Johnson DE, Vogel VG, Johnson CS, Trump DL, Lee YJ, Singh SV (2004). Diallyl trisulfide-induced apoptosis in human prostate cancer cells involves c-Jun N-terminal kinase and extracellular-signal regulated kinase-mediated phosphorylation of Bcl-2. Oncogene.

[CR68] Xiao D, Lew KL, Kim YA, Zeng Y, Hahm ER, Dhir R, Singh SV (2006). Diallyl trisulfide suppresses growth of PC-3 human prostate cancer xenograft in vivo in association with Bax and Bak induction. Clin Cancer Res.

[CR69] Yadav PK, Yamada K, Chiku T, Koutmos M, Banerjee R (2013). Structure and kinetic analysis of H2S production by human mercaptopyruvate sulfurtransferase. J Biol Chem.

[CR70] Yang JB, Wei DY, Wu ZY, Xu SH (2012). DATS suppresses invasion of oral squamous cell carcinoma cel lines by reducing matrix metalloproteinase-9 via PI3 K/AKT. Turk J Biol.

[CR71] Zeng T, Zhang CL, Zhu ZP, Yu LH, Zhao XL, Xie KQ (2008). Diallyl trisulfide (DATS) effectively attenuated oxidative stress-mediated liver injury and hepatic mitochondrial dysfunction in acute ethanol-exposed mice. Toxicology.

[CR72] Zhang F, Zhang Y, Wang K, Liu G, Yang M, Zhao Z, Li S, Cai J, Cao J (2016). Protective effect of diallyl trisulfide against naphthalene-induced oxidative stress and inflammatory damage in mice. Int J Immunopathol Pharmacol.

[CR73] Zhao Y, Biggs TD, Xian M (2014). Hydrogen sulfide (H_2_S) releasing agents: chemistry and biological applications. Chem Commun (Camb).

[CR74] Zhou C, Mao XP, Guo Q, Zeng FQ (2009). Diallyl trisulphide-induced apoptosis in human melanoma cells involves downregulation of Bcl-2 and Bcl-xL expression and activation of caspases. Clin Exp Dermatol.

